# W–Cu Composite with High W Content Prepared by Grading Rounded W Powder with Narrow Particle Size Distribution

**DOI:** 10.3390/ma15051904

**Published:** 2022-03-03

**Authors:** Zheng Chen, Bingxue Li, Qiao Zhang, Xudong Hu, Yi Ding, Zhixiang Zhu, Peng Xiao, Shuhua Liang

**Affiliations:** 1 School of Materials Science and Engineering, Xi’an University of Technology, Xi’an 710048, China; libingxuexx@163.com (B.L.); zhangqiao@xaut.edu.cn (Q.Z.); 2200121114@stu.xaut.edu.cn (X.H.); xiaopeng@xaut.edu.cn (P.X.); 2 Shaanxi Province Key Laboratory of Electrical Materials and Infiltration Technology, Xi’an University of Technology, Xi’an 710048, China; 3 State Key Laboratory of Advanced Power Transmission Technology, Global Energy Interconnection Research Institute Co. Ltd., Beijing 102209, China; dyadin@sina.com (Y.D.); zhuzhixiang003@163.com (Z.Z.)

**Keywords:** W–Cu composite, high W content, gradation, jet milling, infiltration

## Abstract

In this study, the W (10–20%)–Cu composites were simultaneously fabricated using commercial, graded commercial, and graded jet-milled W powder. The results show that the W–Cu composites prepared with the graded jet-milled W powders have the highest density and best comprehensive performance due to the combined effect of the particle gradation and jet-milling treatment. Particle gradation is employed to increase the packing density of powders, thereby increasing the relative density of the compressed W skeleton, and the rounded powder with narrow particle size distribution after jet-milling treatment is used to reduce the enclosed pores formed during the process of compacting and infiltration. W–Cu composites with a high density of 16.25 g/cm^3^ can be directly obtained by conventional compacting at a low pressure of 300 MPa and following infiltration.

## 1. Introduction

Due to its excellent arc erosion resistance, high thermal conductivity, low thermal expansion, and superior mechanical properties, tungsten-copper (W–Cu) composite is widely used in high-voltage circuit-breakers, heat sink materials, shaped charge liners, etc. [1,2,3,4,5,6]. For heat sink materials, the W content of the W–Cu composite is 80–90 wt%, due to the requirement to match the thermal expansion coefficient of the substrate materials. Liquid phase sintering and infiltration are usually used to prepare W–Cu composite [2,4,6,7,8,9,10,11,12,13,14,15]. However, it is hard to fabricate W–Cu composite with a high W content (>80%) by liquid phase sintering because W and Cu are not mutually soluble. For the preparation of W–Cu composite with a high W content by infiltration, there are two problems that need to be solved. First, during the conventional method, mold pressing, it is hard to obtain a W skeleton with a high relative density, which is a prerequisite for the preparation of W–Cu composite with a high W content by infiltration [7,9]. However, the additional sintering process makes it difficult to control the shrinkage of the compacts, which affects the accuracy of the W–Cu product. Second, during the sintering process, the small particles of W powders block the connected channels, forming enclosed pores due to its high sintering activity. Therefore, Boji et al. used Ni and Co as sintering activators to decrease the sintering temperature of the W skeleton by 750 and 560 °C, respectively, and successfully prepared W–10Cu composite by infiltration [7]. Zhou et al. fabricated W–10Cu composite with high relative density via hot-shock consolidation under a relatively low sintering temperature (less than 1000 °C) and a short sintering time (less than 3 min) without any sintering activator [16]. Liu et al. proposed a process to produce W–10Cu composite by liquid phase sintering using nano-sized W–Cu powder with high sintering activity [17]. However, these methods have some limitations, e.g., mechanical property degradation, small geometry, or low productivity.

In powder metallurgy, the relative density of the green compacts is dominated by the packing density and flowability of the powder, which are directly determined by the particle size, particle size distribution, morphology, and agglomeration. Generally, the higher the bulk density and flowability of the powder, the higher the relative density of the green compacts obtained under the same pressure. Powder gradation can improve the bulk density of powder because the pores of large particles can be filled by small particles [18]. German concluded that bimodal powder mixtures can improve the green density of powder systems and are used in the process such as slip casting and powder injection molding [18]. Zou et al. reported that W–30Cu composite prepared by grading W powder has a homogenous microstructure and higher hardness and electrical conductivity [19]. However, the raw material used in this research was commercial W powder, which has a wide particle size distribution and a large number of agglomerated particles. Jet-milling is a powder processing technology that uses high-speed gas to drive powder particles to collide and crush each other, achieving the de-agglomeration, shaping, and classification of powder [20,21,22,23,24,25]. However, there is no report on the preparation of W–Cu composite by particle gradation using jet-milling powder.

In the present study, monodisperse and rounded powders with narrow particle size distribution and high flowability were obtained after jet-milling treatment. Then, W–Cu composites with high W content (80–90 wt%) were prepared by mixing jet-milled W powders with a different particle size in a certain proportion. The microstructure and physical and mechanical properties were investigated, indicating that the graded jet-milled W powder is more suitable for preparing W–Cu composites with a high W content.

## 2. Materials and Methods

### 2.1. Tungsten Powder Treatment

Commercially available W powders with a mean particle size of 3, 5, 8, and 20 μm (designed WY–030, WY–050, WY–080, and WY–200, respectively) were purchased from Xiamen Golden Egret Special Alloy Co. Ltd. (Xiamen, China). The W powders with a mean particle size of 3, 8, and 20 μm needed to be processed by jet-milling, which was conducted in high-purity nitrogen by a QLMR-150T fluidized bed jet-mill (Jilin XDK Electromechanical Technology Co. Ltd., Jilin, China) equipped with a force vortex classifier (designed as WM–030, WM–080, and WM–200, respectively). Then, commercial and jet-milled W powders with a mean particle size of 3, 8, and 20 μm were mixed with a mass ratio of 1:15:15.

### 2.2. W–Cu Composite Preparation

Three kinds of W–Cu composites (W–10Cu, W–15Cu, and W–20Cu) were prepared by infiltration, as listed in Table 1. W powder and traces of Cu powder with a particle size of ~50 μm were mixed in a V-type mixer (Wuxi Jialong Equipment Technology Co. Ltd., Wuxi, China) for 4 h. A certain weight of the blended powders was then pressed to form cylindrical compacts with dimensions of Φ21 × 10 mm and a target relative density under a pressure of 300 MPa. Then, the compacts were sintered and infiltrated at 1350 °C for 2 h using an oxygen-free copper block.

### 2.3. Characterization

The particle size distribution of the W powders was measured on a HELOS-OASIS laser particle size analyzer (SYMPATEC GmbH, Clausthal-Zellerfeld, Germany). The density distribution ($q^{*}(x_{i})$) was calculated by $q^{*}(x_{i})=dQ^{*}(x_{i})/dx_{i}$, where $x_{i}$ is the particle size and $Q^{*}(x_{i})$ is the cumulative distribution). Scanning electron microscopy (SEM) was performed on a Phenom Pure Plus (Phenom, Eindhoven, Netherland) and a JEM-6700F (JEOL, Tokyo, Japan) for W powders and W–Cu composites, respectively. The Archimedes method was used to measure the density of the infiltrated compacts. The Brinell hardness was evaluated with a load of 750 kg and dwell time of 30 s by a HB-2000 tester (Laizhou Huayin Testing Instruments Co. Ltd., Laizhou, China). The electrical conductivity was measured by an Eddy Current Conductivity Meter (Xiamen Tianyan Instrument Co. Ltd., Xiamen, China) and expressed as the International Annealed Copper Standard (IACS) value. At least 5 readings were obtained from the infiltration sample and the average results were calculated while excluding the highest and the lowest readings as the Brinell hardness and the electrical conductivity. Three-point bending tests were conducted at room temperature using a universal materials machine (Instron ElectroPuls E1000, Norwood, MA, USA) at a speed of 0.5 mm/min, and three samples were tested for each W–Cu composite.

## 3. Results

### 3.1. W Powder Treatment

**Figure 1:**
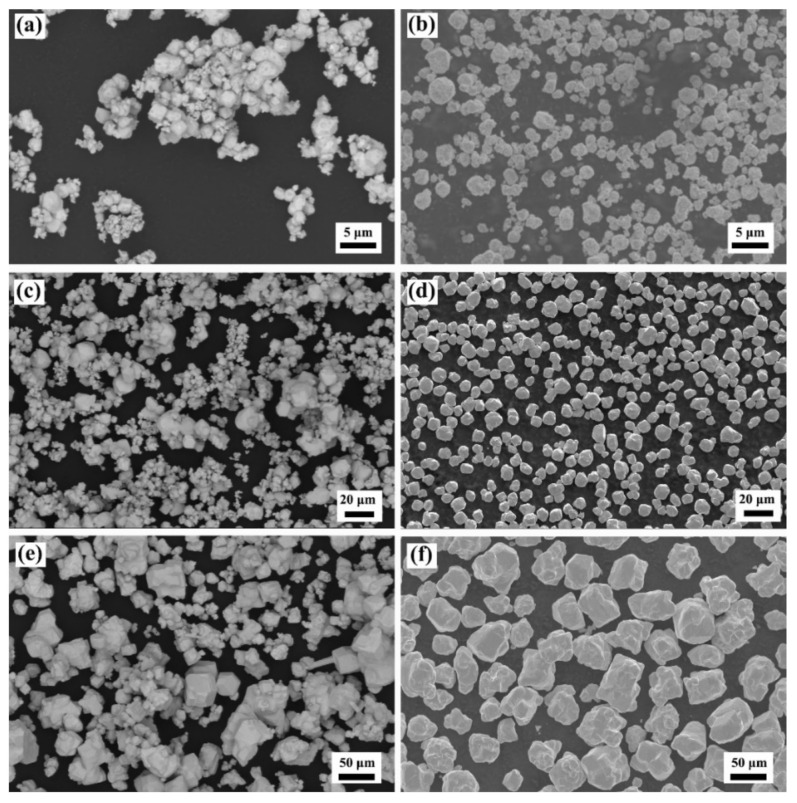
Morphology of commercial (**a**) 3, (**c**) 8, and (**e**) 20 μm and jet-milled (**b**) 3, (**d**) 8, and (**f**) 20 μm W powders.

**Figure 2:**
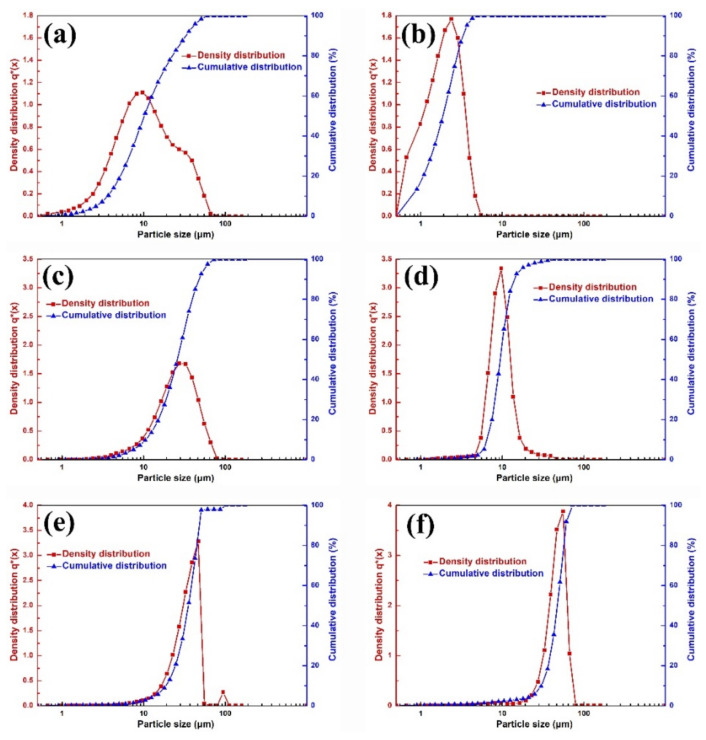
Particle size distribution of commercial (**a**) 3, (**c**) 8, and (**e**) 20 μm and jet-milled (**b**) 3, (**d**) 8, and (**f**) 20 μm W powders.

**Figure 3:**
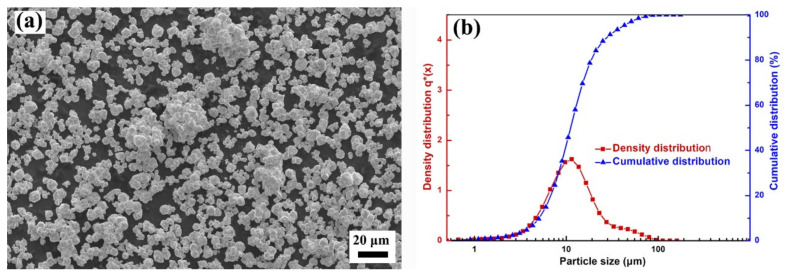
(**a**) Morphology and (**b**) particle size distribution of the WY–050 powder.

Table 2 and Figure 1 show the particle size distribution and morphology of the commercial and jet-milled W powder. The agglomeration of the commercial W powders could be effectively disaggregated by jet-milling. As shown in Figure 1a–c, there were no agglomerated particles in the jet-milled W powders, and WM–050, WM–080, and WM–200 had good dispersibility. The morphology of the W powders changed from the polyhedral shape of the commercial W powders to the rounded shape of the jet-milled W powders. As shown in Figure 2, the particle size distribution of the jet-milled W powders was much narrower than that of the commercial W powders. In detail, the *D*_10_, *D*_50_, *D*_90_, surface mean diameter, and volume mean diameter values of the WM–030 and WM–080 powders were much lower than those of the WY–030 and WY–080 powders, indicating the excellent de-agglomeration effect of the jet-milling process. For the WM–200 powder, the *D*_10_, *D*_50_, *D*_90_, surface mean diameter, and volume mean diameter values were higher than those of the WY–200 powder, suggesting that the jet-milling process can remove the majority of the fine particles. The removal of most of the fine particles is based on the theory of centrifugal grading. In the classifier chamber of jet-milling equipment, the particles are simultaneously subjected to gas flow drag and rotation centrifugal forces. The centrifugal force is proportional to particle weight and the rotating speed of the classification wheel. Eventually, by adjusting the speed, the fine particles are carried out of the classifier chamber by the air flow, and the coarse particles slides back to the grinding chamber. Here, the span value, Ψ, was introduced to evaluate the particle size distribution and can be expressed as $\Psi \;=\;\left(D_{90}-D_{10}\right)/2D_{50}$. As listed in Table 2, the Ψ values of the jet-milled W powders were significantly lower than those of the commercial W powders, which further confirms the narrow particle size distribution of the jet-milled W powders. Figure 3 shows the morphology and particle size distribution of the WY–050 powder. As shown, the WY–050 powder had a large number of agglomerated particles and a wide particle size distribution.

**Table 2:** Particle size distribution of the commercial and jet-milled W powders.

Sample	*D*_10_ (μm)	*D*_50_ (μm)	*D*_90_ (μm)	Ψ	Surface Mean Diameter (μm)	Volume Mean Diameter (μm)
WY–030	3.63	10.23	33.23	1.44	7.20	14.53
WM–030	0.80	1.88	3.32	0.67	1.52	1.98
WY–050	5.26	11.24	27.26	1.96	9.29	14.67
WY–080	10.70	25.95	48.20	0.72	19.24	27.93
WM–080	6.49	9.48	14.21	0.41	8.94	10.30
WY–200	18.78	35.53	48.45	0.42	26.33	35.24
WM–200	30.22	47.41	60.38	0.32	31.07	46.12

### 3.2. Microstructure

**Figure 4:**
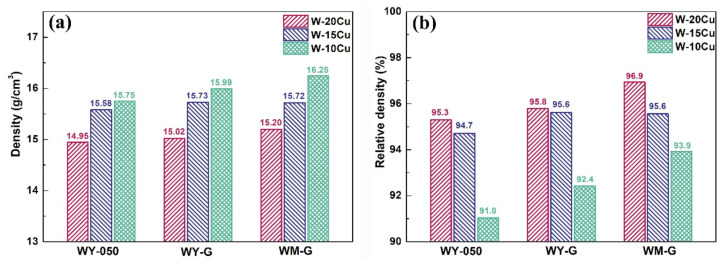
(**a**) Density and (**b**) relative density of the as-prepared W–Cu composites.

Figure 4 and Table 3 show the density and relative density of the as-prepared W–Cu composites. The density and relative density of the as-infiltrated W–Cu composites rose with the increase in the designed Cu content (Figure 4a,b). When the Cu content was identical, the relative density of the W–Cu composites prepared with WM–G powders was the largest compared to those prepared with the WY–050 and WY–G powders. The relative density of the WM–G–10Cu composite reached 93.9%.

**Figure 5:**
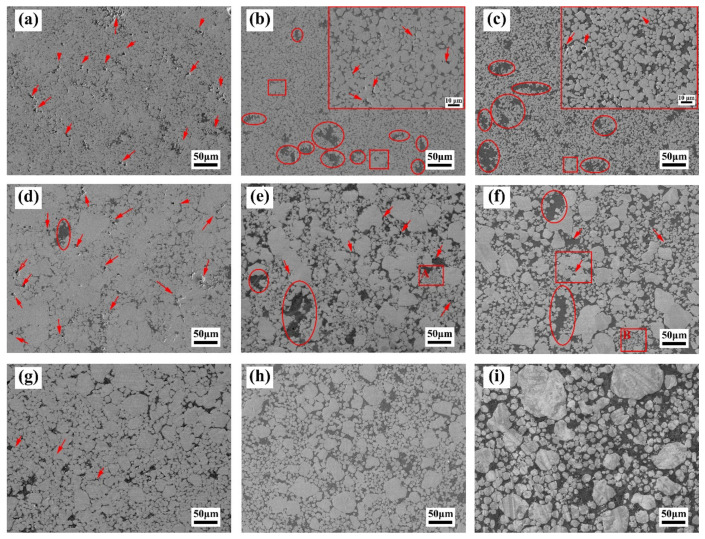
Microstructure of the (**a**) WY–050–10Cu, (**b**) WY–050–15Cu, (**c**) WY–050–20Cu, (**d**) WY–G–10Cu, (**e**) WY–G–15Cu, (**f**) WY–G–20Cu, (**g**) WM–G–10Cu, (**h**) WM–G–15Cu, and (**i**) WM–G–20Cu composites. The red arrows indicate the enclosed pores; the red circles indicate the Cu-rich regions; the red rectangles indicate the agglomerated W particles.

Figure 5 shows the microstructure of the as-prepared W–Cu composites. As shown in Figure 5a–c, a large number of pores (indicated by red arrows) was observed in the WY–050–10Cu composite, and as the Cu content increased, the number of pores decreased. Many Cu-rich regions (indicated by red circles) and agglomerated W particles (indicated by red rectangles) were present as well. Here, the Cu-rich region was defined as an “isolated” Cu phase with a size significantly higher than that of the W particles, in which no W particles are present. As for the WY–G–Cu composites, the Cu-rich regions and agglomerated W particles decreased significantly, but the number of pores was still large. As shown in Figure 5e,f, large W particles (indicated by A and B) aggregated by ultrafine particles were observed in the WY–G–15Cu and WY–G–20Cu composites, which might have originated from the agglomerated particles in the WY–030 and WY–050 powders (Figure 1a and Figure 2a). This was especially the case for the WY–G–10Cu composite because the W particles were almost all clustered together. This led to the rapid sintering of the W particles and the closure of the pores; a large number of pores is shown in Figure 5d. However, there were almost no pores in the WM–G–15Cu and WM–G–20Cu composites (Figure 5h,i), but a small number of pores was still observed in the WM–G–10Cu composite (Figure 5g). On the other hand, the W and Cu phases of the WM–G–Cu composites were uniformly distributed, and there were no apparent Cu-rich regions and agglomerated W particles. The microstructure of the W–Cu composites did not match its relative density; that is, the relative density observed in Figure 5 was significantly higher than the measured value. From the results, the actual Cu content of the W–Cu composites was higher than the design value. To summarize, the W–Cu composites prepared by grading rounded W powder with narrow particle size distribution had the highest actual density compared to those fabricated using the commercial powders.

### 3.3. Physical Properties

**Figure 6:**
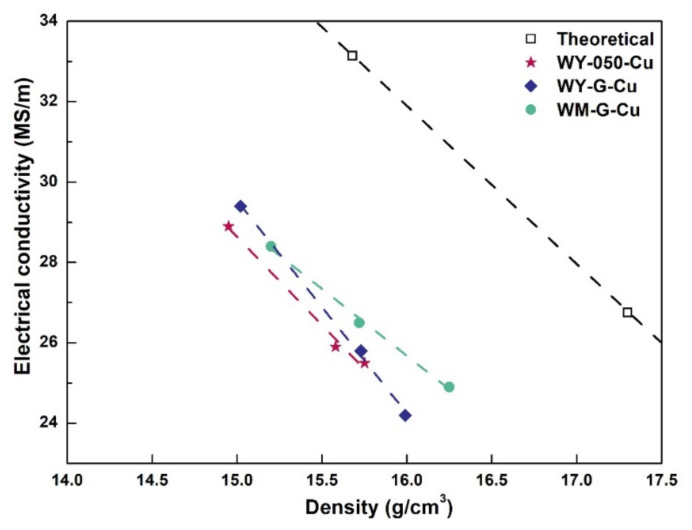
The electrical conductivity vs. density of the as-prepared W–Cu composites.

The electrical conductivity of the W–Cu s is listed in Table 3. It is shown that the electrical conductivity decreased with the increase in the W content for all W–Cu composites. The electrical conductivity of the W–Cu composites is directly determined by the Cu content. However, it is difficult to accurately prepare W–Cu composites with a specific Cu content because the relative density of the W skeleton for infiltration cannot be accurately controlled and the amount of the Cu loss during the liquid phase sintering process cannot be estimated. Therefore, it is hard to analyze small discrepancies in the electrical conductivity of W–Cu composites with an identical Cu content by the difference in W powder. Figure 6 shows the electrical conductivity and density trajectories of the as-prepared W–Cu composites. The electrical conductivity of the WY–050–Cu and WY–G–Cu composites decreased rapidly with the increase in the density. Although the electrical conductivity of the WM–G–Cu composites also decreased as the density increased, the rate of decline was much slower. That is, the W–Cu composites prepared by the WM–G powder had both higher density and higher electrical conductivity.

### 3.4. Mechanical Properties

Normally, the Brinell hardness of W–Cu composites decreases with the increase in Cu content. As listed in Table 3, the Brinell hardness of the WY–G–Cu and WM–G–Cu composites increased from ~210 HB of the W–20Cu composites to ~230 HB of the W–10Cu composites. Anomalously, the Brinell hardness of the WY–050–15Cu composite was not only higher than that of the WY–050–20Cu composite, but also higher than that of the WY–050–10Cu composite. This may be ascribed to the large number of pores in the WY–050–10Cu composite, which led to the decrease in hardness. The hardness of the WY–050–Cu composites was much higher than those of the WY–G–Cu and WM–G–Cu composites with the same Cu content due to the small size of the W particles in the WY–050 powder.

**Figure 7:**
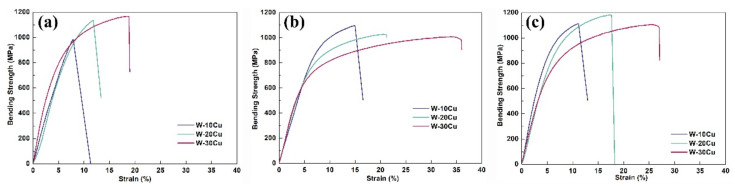
Bending strength-strain curves of the as-prepared W–Cu composites using (**a**) WY–050, (**b**) WY–G, and (**c**) WM–G powders.

**Figure 8:**
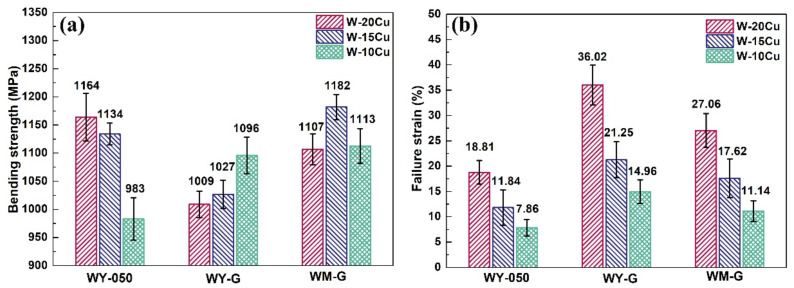
(**a**) Bending strength and (**b**) failure strain of the as-prepared W–Cu composites.

Figure 7 and Figure 8 show the bending strength–strain curves, bending strength, and failure strain of the as-prepared W–Cu composites. For W–20Cu composite, the WY–050–20Cu composite had the highest bending strength, 1164 MPa, and the bending strength of the WY–G–20Cu composite was only 1009 MPa, lower than the 1107 MPa of the WM–G–20Cu composite. The bending strength of the WM–G–15Cu and WM–G–10Cu composites was higher than those of the WY–050–Cu and WY–G–Cu composites with an identical Cu content. The bending strength of the WM–G–15Cu and WM–G–10Cu composite reached 1182 and 1113 MPa, respectively. However, the bending strength of the WY–050–10Cu was only 983 MPa. As shown in Figure 8b, the failure strain decreased with the increase in W content for all W–Cu composites, whereas compared to the WY–050–Cu and WM–G–Cu composites, the WY–G–Cu composites had the highest failure strain with the same Cu content. The failure strain of the WM–G–20Cu, WM–G–15Cu, and WM–G–10Cu composites reached 27.06%, 17.62%, and 11.14%, respectively, which are lower than those of the WY–G–Cu composites. According to Figure 7 and Figure 8, the bending strength of the WY–050–Cu composites was higher than those of the WY–G–Cu composites, but the failure strain was much lower. In contrast, although the failure strain of the WY–G–Cu composites was the highest, its bending strength was the lowest. Hence, the WM–G–Cu composites had the best comprehensive bending performance.

**Figure 9:**
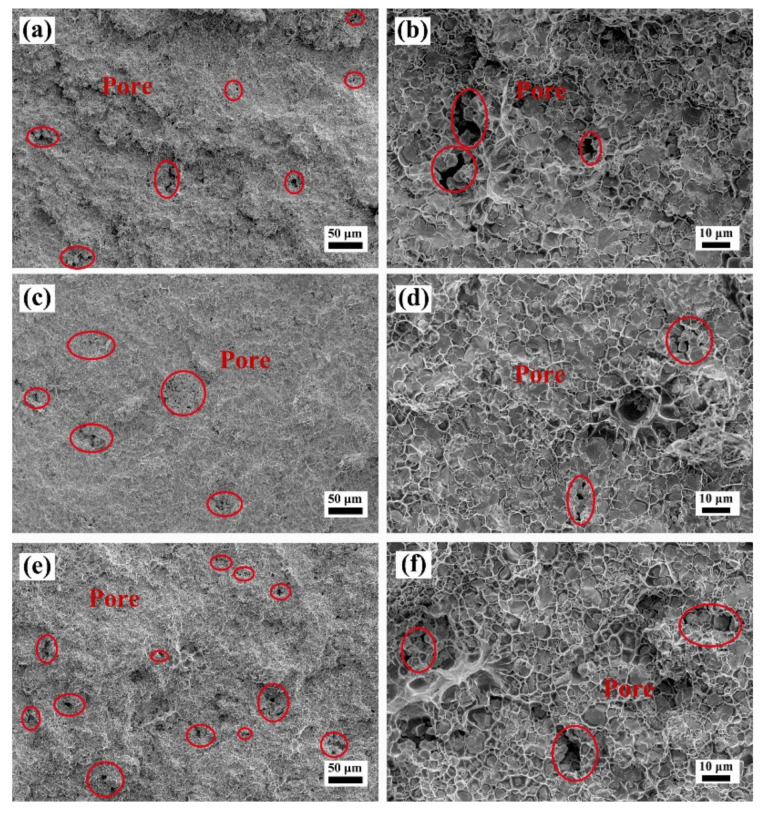
SEM images of the fracture surface of the (**a**,**b**) WY–050–10Cu, (**c**,**d**) WY–050–15Cu, and (**e**,**f**) WY–050–20Cu composites after bending tests.

**Figure 10:**
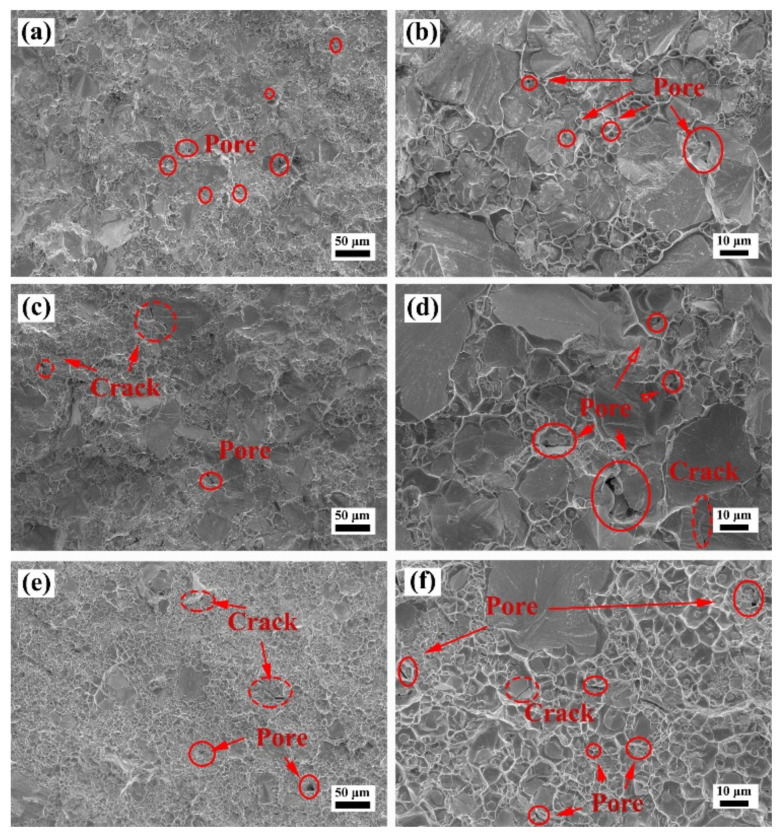
SEM images of the fracture surface of the (**a**,**b**) WY–G–10Cu, (**c**,**d**) WY–G–15Cu, and (**e**,**f**) WY–G–20Cu composites after bending tests.

**Figure 11:**
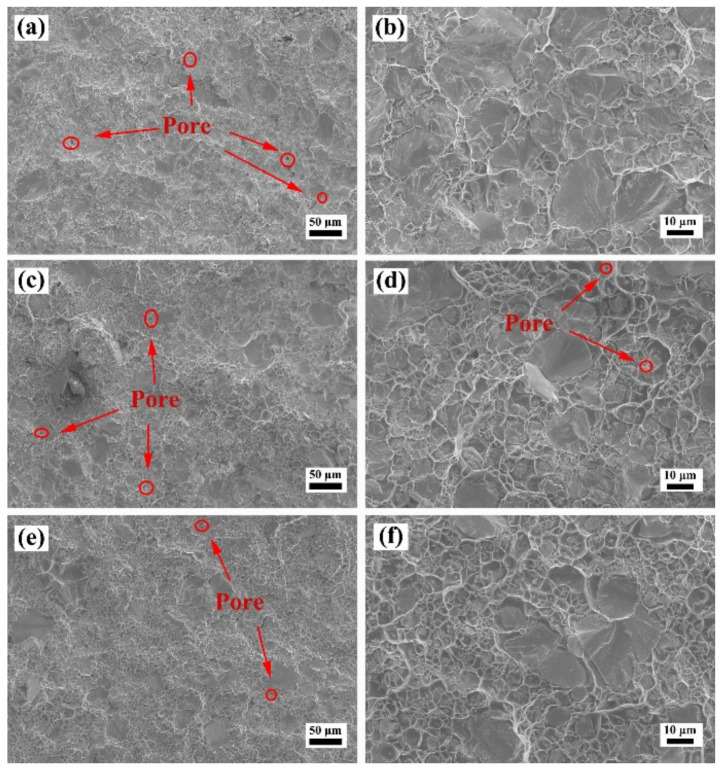
SEM images of the fracture surface of the (**a**,**b**) WM–G–10Cu, (**c**,**d**) WM–G–15Cu, and (**e**,**f**) WM–G–20Cu composites after bending tests.

Figure 9, Figure 10 and Figure 11 show the SEM images of the fracture surface of the W–Cu composites after bending tests. As shown in Figure 9, a large number of pores was found in the fracture surface of the WY–050–Cu composites, and W particle aggregations and Cu-rich regions were also observed. Due to the large amount of ultra-fine particles of WY–050 powder and the extremely high relative density of the green compact, enclosed pores formed in the W skeleton during infiltration, preventing the filling of the liquid Cu phase. Therefore, although the WY–050–Cu composites had the smallest average particle size, which deflected the cracks, the large number of pores was the source of cracks during bending tests, resulting in the relatively high bending strength and ultralow failure strain of the WY–050–Cu composites. However, for the WY–G–Cu composites, there were fewer pores, but many cracked W agglomerated areas were present, as shown in Figure 10. As shown in Figure 10a,b, there was a large number of small pores in the WY–G–10Cu composite, which were also caused by the sintering of the small W particles. As shown in Figure 11, only a few pores were found in the WM–G–Cu composites, which was consistent with the best comprehensive bending performance.

## 4. Discussion

In general, mono-sized spherical powders approach a fraction packing density of 0.64 in a random arrangement, and mixtures of powders with different sizes have improved packing densities [26]. In this study, the packing density of the WY–050, WY–G and WM–G powders were 4.63, 6.96, and 6.98 g/cm^3^, respectively. The packing density of the graded powders increased by more than 50% compared to that of the ungraded powder, which significantly benefitted the improvement of the relative density of green compacts.

**Figure 12:**
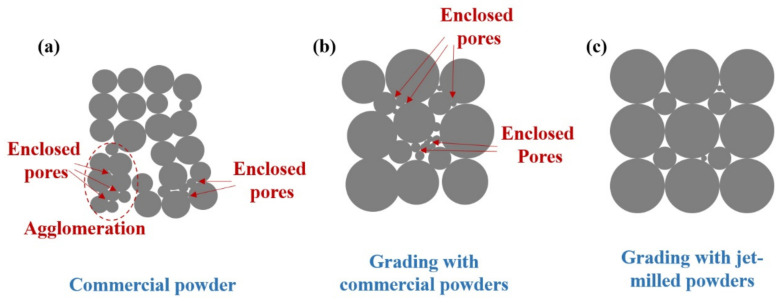
Schematic diagram of the accumulation of (**a**) WY–050, (**b**) WY–G, and (**c**) WM–G powders.

As shown in Figure 12a, due to the bridge and polygonal morphology, it was difficult for the WY–050 powder with a normal particle size distribution to produce a W skeleton with high relative density. There were many agglomerations and fine particles in the WY–050 powder, which led to the formation of a large number of enclosed pores during the compacting and infiltration process. As for the WY–G powder, small- particles filled the gaps in the large particles, thereby increasing the compaction density of the W skeleton. However, a large number of enclosed pores caused by agglomerations and ultrafine particles still existed (Figure 12b). In this study, we not only used jet-milling for de-agglomeration, but also separated and collected the W powders with different particle sizes through the classification device of the jet-milling equipment based on the difference in the centrifugal force of the different particle size powders. In the WM–G powder, rounded W powders with narrow particle size distribution were mixed and the ultrafine particles were removed. Therefore, the compaction density of the WM-G powers increased, but enclosed pores rarely appeared.

In this study, we intended to obtain a W–Cu composite with a designed Cu content by controlling the relative density of the W skeleton. However, due to the limitations, only a pressure of 300 MPa could be used when pressing the W skeleton. Therefore, the relative density of the W skeleton did not all reach the designed value. For the preparation of the W–15Cu and W–20Cu composites, the WY–050–Cu, WY–G–Cu and WM–G–Cu all reached the desired relative density by controlling the height of the green compacts. However, combining the results of Table 3 and Figure 5, it can be seen that the Cu content in W–10Cu composites was obviously greater than 10 wt%, suggesting that the relative density of the W skeleton had not reached the desired value. Therefore, even if there were no obvious pores found in the WM–G–10Cu composite, the relative density was only 93.9%, which was still significantly higher than the 91.6% and 92.4% of the WY–050–10Cu and WY–G–10Cu composites, respectively.

In order to obtain actual W–10Cu composite, pressing with higher pressure or an additional high-temperature sintering process needs to be conducted [9,27]. However, a large number of enclosed pores was observed in the WY–050–10Cu and WY–G–10Cu composites, and the larger green density caused the number of pores to continue to increase, which severely deteriorated the conductivity of the W–Cu composites. Herein, the jet-milling process was not only used for de-agglomeration, but also to separate and collect the W powders with different particle sizes through the classification device of the jet-milling equipment based on the difference in the centrifugal force of the powders with different particle sizes. Therefore, in the WM–G powder, the fine particles were almost completely removed. It is rare for fine particles to block channels to form closed pores during the pressing or high-temperature sintering process. Hence, using the WM–G powder not only can obtain the W skeleton with higher relative density due to the high packing density, but also does not generate a large number of closed pores resulting from the increase in density. A low pressure of 300 MPa was achieved by the cold isostatic pressing process, suggesting the possibility of using the WM–G powder to prepare large W–Cu composites.

## 5. Conclusions

In this study, W (10–20%)–Cu composites were prepared using grading rounded W powder with narrow particle size distribution treated by a jet-milling process. The microstructure and physical and mechanical properties were investigated. The following conclusions were drawn from the presented work:

1. Rounded W powders with narrow particle size distribution are obtained by jet-milling.
2. W–Cu composites prepared with graded powder have higher density. W–Cu composite with a high density of 16.25 g/cm^3^ can be directly obtained by conventional compacting at a low pressure of 300 MPa and following infiltration using graded W powders, which are treated through jet-milling.
3. There are few pores in WM–G–10Cu composite, but a large number of pores in WY–050–10Cu and WY–G–10Cu composites. The WM–G–Cu composites have the best comprehensive performance.
4. WM–G powder is more suitable for preparing W–Cu composites with a high W content and more likely to be used to obtain large W–Cu composites.

## Figures and Tables

**Table 1:** Mass ratio of different W powders in the gradation.

Sample	Commercial W Powder (wt%)				Jet-Milled W Powder (wt%)			Cu (wt%)
	3 μm	5 μm	8 μm	20 μm	3 μm	8 μm	20 μm	
WY–050–20Cu	-	80	-	-	-	-	-	20
WY–050–15Cu	-	85	-	-	-	-	-	15
WY–050–10Cu	-	90	-	-	-	-	-	10
WY–G–20Cu	2.58	-	38.71	38.71	-	-	-	20
WY–G–15Cu	2.74	-	41.13	41.13	-	-	-	15
WY–G–10Cu	2.90	-	43.55	43.55	-	-	-	10
WM–G–20Cu	-	-	-	-	2.58	38.71	38.71	20
WM–G–15Cu	-	-	-	-	2.74	41.13	41.13	15
WM–G–10Cu	-	-	-	-	2.90	43.55	43.55	10

**Table 3:** Density, electrical conductivity, and hardness of the as-prepared W–Cu composites.

Sample	Density (g/cm^3^)	Relative Density (%)	Electrical Conductivity		Hardness (HB)
			MS/m	%IACS	
WY–050–20Cu	14.95 ± 0.04	95.3 ± 0.26	28.9 ± 0.10	49.8 ± 0.16	229 ± 6.66
WY–050–15Cu	15.58 ± 0.03	94.7 ± 0.16	25.9 ± 0.02	44.7 ± 0.03	248 ± 7.51
WY–050–10Cu	15.75 ± 0.04	91.0 ± 0.23	25.5 ± 0.05	44.0 ± 0.08	241 ± 5.57
WY–G–20Cu	15.02 ± 0.03	95.8 ± 0.17	29.4 ± 0.11	50.7 ± 0.19	209 ± 4.04
WY–G–15Cu	15.73 ± 0.04	95.6 ± 0.25	25.8 ± 0.05	44.5 ± 0.08	224 ± 2.08
WY–G–10Cu	15.99 ± 0.09	92.4 ± 0.51	24.2 ± 0.18	41.7 ± 0.32	231 ± 5.51
WM–G–20Cu	15.20 ± 0.04	96.9 ± 0.23	28.4 ± 0.16	45.0 ± 0.27	211 ± 8.33
WM–G–15Cu	15.72 ± 0.03	95.6 ± 0.18	26.5 ± 0.03	45.7 ± 0.05	222 ± 2.52
WM–G–10Cu	16.25 ± 0.04	93.9 ± 0.24	24.9 ± 0.14	42.9 ± 0.24	232 ± 5.51

## Data Availability

The data presented in this study are available on request from the corresponding author.
